# Optical Control of Adenosine A_3_ Receptor Signaling: Towards a Multimodal Phototherapy in Psoriasis?

**DOI:** 10.3389/fimmu.2022.904762

**Published:** 2022-04-29

**Authors:** Francisco Ciruela, Kenneth A. Jacobson

**Affiliations:** ^1^Pharmacology Unit, Department of Pathology and Experimental Therapeutics, Faculty of Medicine and Health Sciences, Institute of Neurosciences, University of Barcelona, L’Hospitalet de Llobregat, Barcelona, Spain; ^2^Neuropharmacology and Pain Group, Neuroscience Program, Institut d’Investigació Biomèdica de Bellvitge, IDIBELL, L’Hospitalet de Llobregat, Barcelona, Spain; ^3^Molecular Recognition Section, Laboratory of Bioorganic Chemistry, National Institute of Diabetes and Digestive and Kidney Diseases, National Institutes of Health, Bethesda, MD, United States

**Keywords:** psoriasis, phototherapy, adenosine, anti-inflammatory, adenosine A_3_ receptor, photopharmacology

## Introduction 

Psoriasis is a long-lasting inflammatory disease primarily characterized by cutaneous and systemic manifestations but also showing multiple comorbidities (i.e., psoriatic arthritis, cardiometabolic diseases, psychological illnesses, inflammatory bowel diseases), which affect patients’ quality of life. Its global prevalence score fluctuates around 2% of the population, from which 70% to 80% show a mild variant (i.e., less than 3% to 5% of affected body surface area), and is equally present in both sexes (1). Current treatments of psoriasis show excellent clinical efficacy for many patients but are not curative and eventually remain deficient or inefficient for many others. Thus, despite the therapeutic arsenal for psoriasis being considered first-rate, some unmet clinical conditions will require further pharmacotherapeutic development. In that context, novel orally active drugs for the management of moderate-to-severe psoriasis are under development (2), including Piclidenoson (CF101), an adenosine A_3_ receptor (A_3_R) agonist. Indeed, A_3_R has emerged as novel, promising therapeutic target and biologically predictive marker not only for psoriasis but also for other inflammatory diseases (i.e., rheumatoid arthritis) (3).

**Figure 1:**
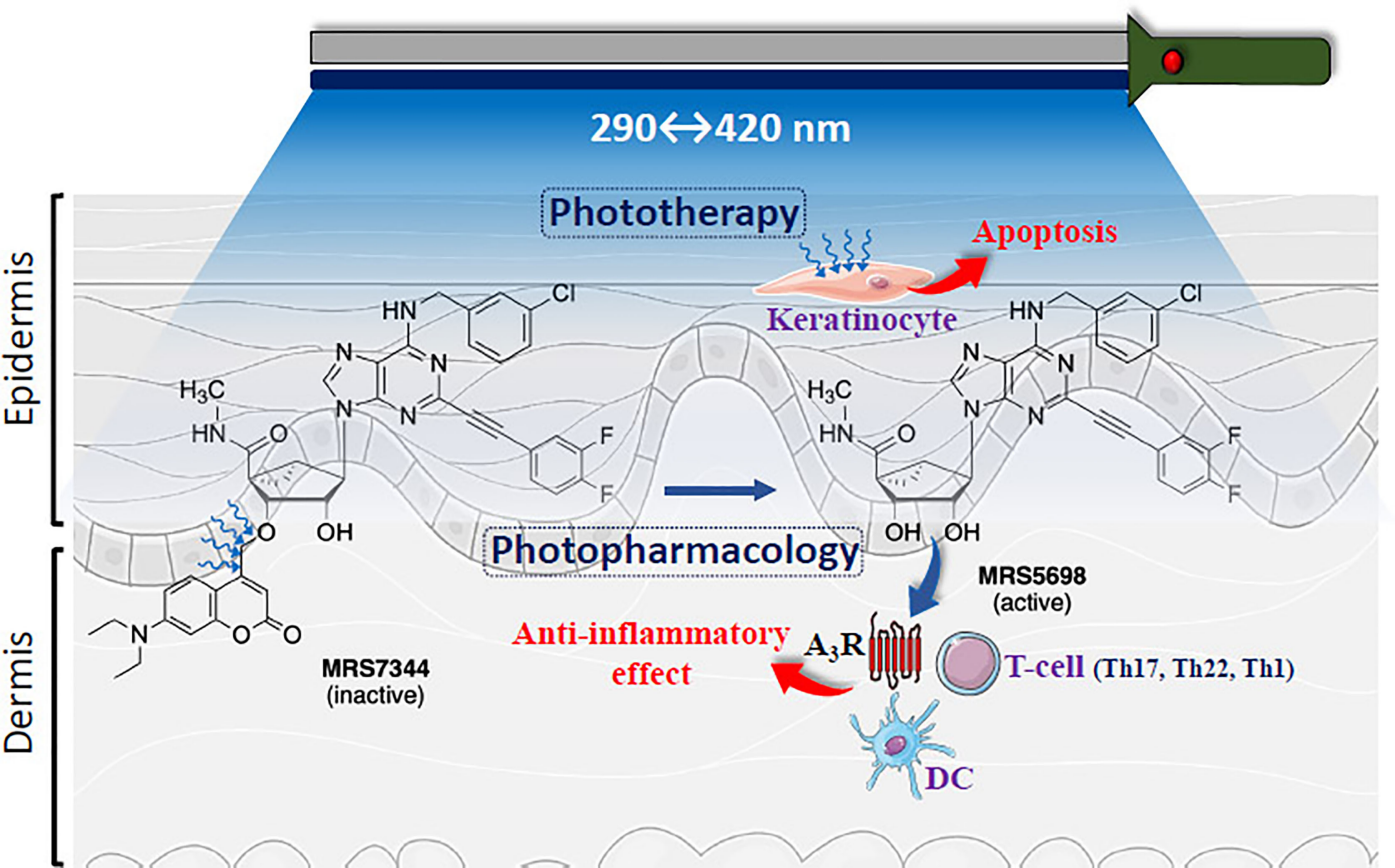
Schematic representation of multimodal phototherapy. Cartoon showing skin layers (i.e., epidermis and dermis) being light irradiated. The structures of highly potent and selective A_3_R agonist MRS5698 and its photocleavable prodrug MRS7344, which is masked as an ether at the 3’-hydroxyl group with a 7-diethylamino-4-hydroxymethylcoumarin (DEAC) moiety is depicted. UV-based light irradiation (290-400 nm) will induce keratinocyte apoptosis (i.e., Phototherapy). In addition, photorelease of MRS5698 with 420 nm may potentially activate A_3_R within immune cells, including T cells (CD8^+^, Th17, Th22, Th1) and dendritic cells (DC) (5), thus promoting anti-inflammatory effects and alleviating psoriasis. Figure designed using image templates from Servier Medical Art (https://smart.servier.com/image-set-download/).

Photopharmacology is a general approach for using visible light to convert a conformationally inactivate or chemically masked form of a drug molecule to its biologically active form at the site of action in the body (4). Recently, a photocaged A_3_R agonist, MRS7344 (**Figure 1**), which is a photocleavably blocked form of the potent and selective agonist. MRS5698, was reported (6). The full agonist MRS5698 is >3000-fold selective for the A_3_R compared to other adenosine receptors in multiple species and has been shown to be well tolerated *in vivo* (7). Interestingly, in a preclinical model of psoriasis (i.e., IL-23 psoriatic mouse model) MRS7344 demonstrated superior anti-psoriatic-like efficacy in a light-dependent fashion and upon non-toxic non-invasive topical procedure (6). Interestingly, the potential incorporation of this new A_3_R-based photopharmacological solution for psoriasis management may be expanding the already existing clinical therapeutic use of light (i.e., phototherapy). Of note, while preclinical photopharmacology uses photoactivable drugs (i.e., photodrugs) allowing the optical control of specific molecular targets with high spatiotemporal resolution, clinical phototherapy relies on photochemical processes striking endogenous biomolecules not selectively, thus being limited by a lack of specificity (8). Indeed, since both approaches operate with light (**Figure 1**), it appears feasible to find combined light formulations allowing a multimodal treatment of dermatological conditions in general and psoriasis in particular.

## Adenosine Receptors as Anti-Inflammatory Mediators

In general, activation of adenosine receptors produces anti-inflammatory effects (9). In psoriasis, while the A_2A_R and A_2B_R are upregulated and downregulated in keratinocytes, respectively, the A_3_R is overexpressed in peripheral blood mononuclear cells from patients. Activation of adenosine receptors reduces proinflammatory cytokine release from macrophages, dendritic cells (DCs), and T cells (i.e., Th17, Th22, Th1), thus preventing psoriatic cytokines (i.e., IL-17, IL-22, TNF, and IFN-γ) to promote both a positive inflammatory feedback and keratinocyte hyperproliferation [for review see (5, 9)]. Accordingly, activation of adenosine receptors may prove useful in psoriasis. Among the four subtypes of adenosine receptors, A_1_, A_2A_, A_2B_ and A_3_, the A_3_R has the most ongoing clinical trials of agonists (10). In previous studies of A_1_R and A_2A_R agonists, unacceptable cardiovascular side effects were often found, while A_3_R agonists in clinical trials show lack of serious adverse effects. Piclidenoson (CF101), generically known as IB-MECA (methyl 1-[*N*^6^-(3-iodobenzyl)-adenin-9-yl]-β-D-ribofuronamide), is an orally bioavailable active A_3_R agonist currently in an advanced clinical trial (Phase III: NCT03168256) for the treatment of moderate-to-severe plaque psoriasis. While top line data from this study is scheduled to be released in the second quarter of 2022, the preliminary results showed high efficacy compared to apremilast (Otezla®), an oral PDE4 inhibitor, as well as an excellent safety profile, thus suggesting its promise as a chronic treatment (3).

## Will Multimodal Phototherapy be Suitable for Resistant Psoriasis? 

While the recommended management of mild psoriasis is based on topical treatments (i.e., corticosteroids, keratolytics, calcineurin inhibitors and vitamin D analogues), moderate-to-severe psoriasis is generally addressed clinically with systemic approaches (i.e., biologic agents and/or oral medications). In addition, phototherapy (in the absence or presence of systemic treatments) has been successfully used for decades to treat patients with either mild, moderate or severe plaque psoriasis (11).

Compared to sunlight, both targeted (for mild psoriasis) or widespread (for moderate-to-severe psoriasis) phototherapy consists of dosing specific wavelengths that are therapeutic and limiting those that are carcinogenic. Several types of irradiation schedules are used according to the psoriatic condition, for instance, targeted phototherapy of localized plaque psoriasis uses excimer light that emits high-intensity UV-B (i.e., 308 nm). In full-body-surround phototherapy UV-B light, both narrow (i.e., 311 nm) and broad (i.e., 290–320 nm) bandwidths are used. In any case, UV irradiation impedes DNA synthesis to induce keratinocyte apoptosis and reduces production of proinflammatory cytokines by immune cells. In addition, for widespread phototherapy the treatment with a psoralen (i.e., methoxalen) plus UV-A (i.e., 320–400 nm) irradiation (PUVA) may be implemented. While oral PUVA treatment has superior efficacy to UV-B, there is an elevated risk of skin cancer development with long-term use (11). Interestingly, full body and localized UV-based therapies can be administered at home through available phototherapy devices providing safe and effective treatments, as those administered in outpatient setting (12).

Although not very common, psoriasis that is resistant to topical and systemic treatment constitutes a serious clinical problem (12). Indeed, poor patient adherence to anti-psoriatic medications is the main factor responsible for treatment resistance, especially in topical therapies, due to the burden of continuous application and the undesirable aesthetics of creams or greasy ointments (12). Nevertheless, true treatment resistance may eventually occur in patients who develop anti-drug antibodies, especially those treated with a biologic agent, or who are insufficiently dosed or develop treatment tachyphylaxis (12). Strategies to tackle resistance to anti-psoriatic treatments begin by addressing adherence in the first instance before escalating, combining, or switching medications. In patients showing sub-optimal responses to current medication dose escalation is an option, although not without risk (i.e., biologic agents), thus often adding a second anti-psoriatic agent is a common clinical choice. Accordingly, combining in a multimodal fashion different topical and systemic medications with phototherapy increases the management options of psoriasis, particularly for those recalcitrant manifestations of the disease.

In line with this, expanding light-based therapies towards the use of new systemic photoactivable drugs is provocative as it will generate novel strategies within the multimodal treatment algorithm tackling recalcitrant psoriasis. Thus, combining light-compatible systemic A_3_R-based anti-psoriatic photodrugs (i.e., MRS7344) (6) with current phototherapy approaches may increase the efficacy and reduce the adverse effects of these light-based therapies while boosting the selective systemic pharmacotherapy (**Figure 1**). In addition, this multimodal light-based therapy might simplify the treatment regimen by adjusting the dosing to once-daily light administration without decreasing anti-psoriatic efficacy, thus reducing treatment burden, and increasing adherence to the medication. Overall, the possibility of combining photopharmacology and phototherapy in psoriasis (**Figure 1**) sounds promising, especially for those psoriatic conditions with problematic clinical management (i.e., resistant psoriasis).

## Concluding Remarks and Future Directions

Topical therapies remain the cornerstone for mild psoriasis treatment, while systemic treatments are reserved for moderate-to-severe manifestations of the disease, and both being able to combine with phototherapy (11). While most psoriatic conditions are successfully tackled with existing drugs, either in single therapies or multimodal approaches, no treatment algorithm for the effective management of resistant psoriasis exits (12). The advent of novel small molecules for oral administration will increase the chances of generating new stand-alone or combined anti-psoriatic therapies, thus broadening the guidance for recalcitrant psoriasis. In that sense, photoactivable anti-psoriatic selective drugs compatible current phototherapy settings will definitively enable new therapeutic opportunities for psoriasis. Indeed, the well-defined mechanism of action of A_3_R agonists, inducing a T cell-mediated immunosuppressive response in psoriasis, together with the ability to manipulate the intrinsic activity of these ligands with light (i.e., photopharmacology), have generated a potent and tunable pharmacological tool for psoriasis treatment. Finally, these preclinical clues need to be further explored and expanded before any clinical consideration, and certainly, the success in the clinical development of Piclidenoson will greatly facilitate it.

Overall, introducing novel light-activable drugs acting through the A_3_R to locally manipulate the immune system in psoriasis might open avenues to develop novel multimodal phototherapeutic strategies for this common inflammatory skin disorder.

## Author Contributions

FC and KJ wrote the first draft of the manuscript and approved the submitted version.

## Funding

Supported by project PID2020-118511RB-I00 founded by MCIN/AEI/10.13039/501100011033 “ERDF A way of making Europe” and Generalitat de Catalunya(2017SGR1604).

## Conflict of Interest

The authors declare that the research was conducted in the absence of any commercial or financial relationships that could be construed as a potential conflict of interest.

## Publisher’s Note

All claims expressed in this article are solely those of the authors and do not necessarily represent those of their affiliated organizations, or those of the publisher, the editors and the reviewers. Any product that may be evaluated in this article, or claim that may be made by its manufacturer, is not guaranteed or endorsed by the publisher.
